# Supplementary material for: Molecular Dissection of Phagocytosis by Proteomic Analysis in Entamoeba histolytica
Source: Genes (Basel). 2023 Jan 31;14(2):379. doi: 10.3390/genes14020379 (PMC9957367; doi:10.3390/genes14020379)
Supplement: Supplementary file 1 [file genes-14-00379-s001.zip › genes-2148307-Supplemental_figure_Na230124-highlight.pdf]

Supplemental Figure S1

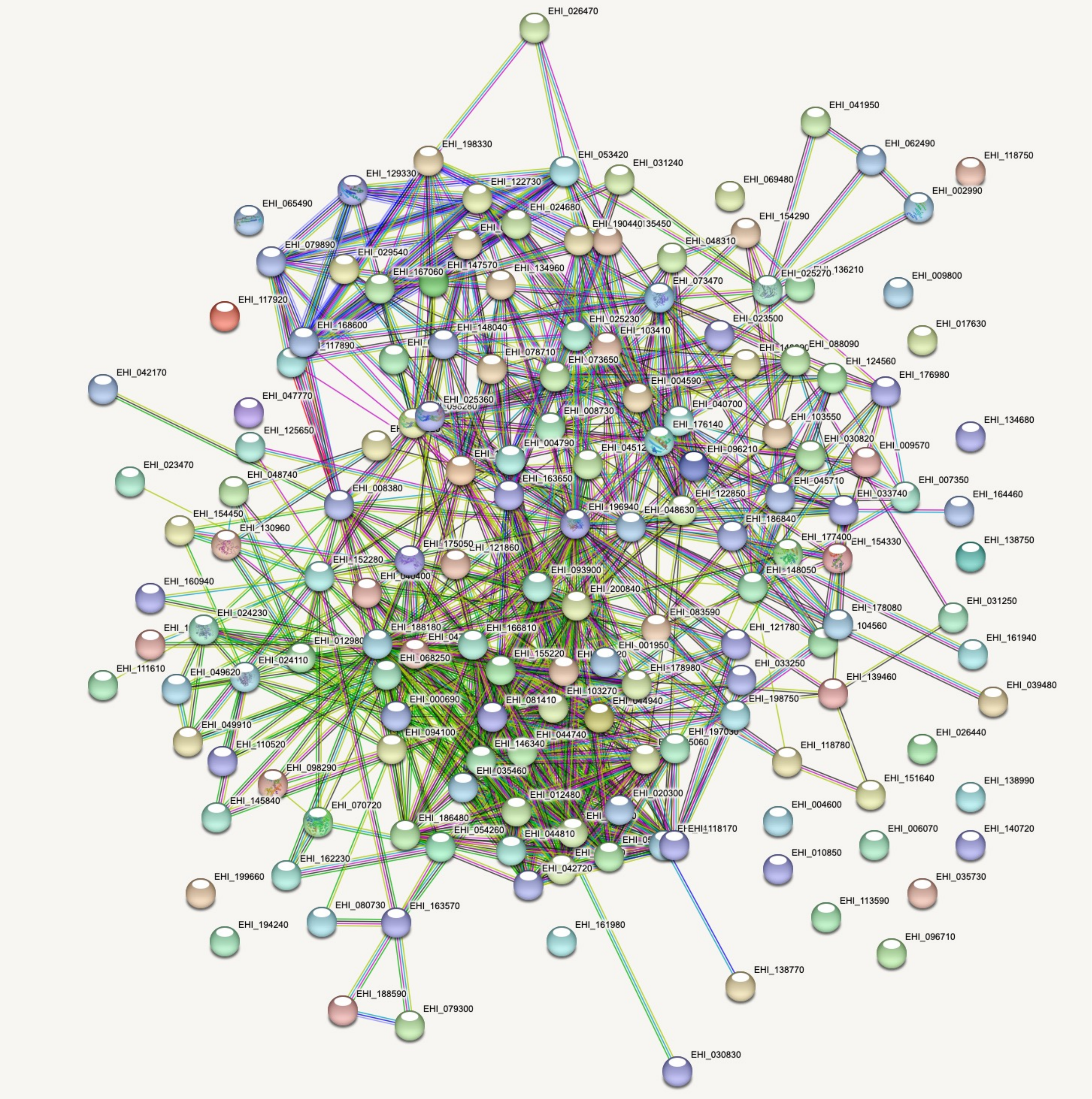

Supplemental Figure S2

Transiently or condition-dependently associated phagosome proteins (magnetic beads)

Stably associated phagosome proteins (density-gradient)

- Small G protein (canonical Rabs)
- Degrading enzyme (gp63, CPs, lysozyme)
- Membrane traffic protein
- Lysosomal enzyme
- Metabolic enzyme
- Actin related protein
- Surface protein

- RNA metabolism protein
- Chaperone
- Chromatin/chromatin-binding protein
- Cytoskeletal protein (actin binding proteins)
- Membrane traffic protein (retromer components)
- Metabolite interconversion enzyme
- Protein modifying enzyme (ubiquitin-proteasome)
- Protein-binding activity modular (*E. histolytica*-specific Rabs)
- Scaffold/adaptor protein
- Transfer/carrier protein
- Translational protein (ER-localized proteins)
- Transmembrane signal receptor (hemolysin)
- Transporter (sec13)
